# Trends in Salt Consumption and Reduction Practices in Vietnam During 2015–2021: Analyzing Urinary Sodium Levels Among 18–69 Aged Populations

**DOI:** 10.3389/ijph.2025.1608065

**Published:** 2025-03-25

**Authors:** Vu Thi Hoang Lan, Bui Thi Tu Quyen, Pham Quang Duy, Le Hoang, Hoang Van Minh

**Affiliations:** ^1^ Faculty of Fundamental Sciences, Hanoi University of Public Health, Hanoi, Vietnam; ^2^ Faculty of Sciences, University of British Columbia, Vancouver, BC, Canada

**Keywords:** sodium, salt intake, non-communicable diseases (NCDs), diabetes and hypertension, high BMI

## Abstract

**Objectives:**

This study investigates changes in salt intake and reduction practices among Vietnamese adults (ages 18–69), focusing on high-risk groups for non-communicable diseases (NCDs) like hypertension, diabetes, and elevated BMI.

**Methods:**

Participants aged 18–69 from the 2015 and 2020 STEPs surveys provided data on the spot urine test. Average daily salt intake was calculated using the Intersalt Southern European equation. The prevalence of excessive salt intake was assessed, along with subgroup analyses based on demographic factors and NCD risk.

**Results:**

Average salt intake decreased significantly from 9.42 g/day in 2015 to 8.07 g/day in 2020 (p < 0.01), with the most substantial decline among younger individuals. The percentage exceeding the global average of 10.78 g/day dropped from 24.88% to 8.31%. High-risk groups, including those with hypertension and diabetes, consumed more salt but also showed reductions. Awareness of salt reduction advice remained low, with only 60.9% of the general population informed.

**Conclusion:**

While progress has been made in reducing salt intake, ongoing public health initiatives are essential to meet recommended levels, especially for high-risk populations.

## Introduction

Excessive salt intake is a well-documented risk factor for numerous non-communicable diseases (NCDs), especially hypertension, cardiovascular diseases, and stroke [1, 2]. Globally, public health efforts are increasingly emphasizing reducing sodium consumption as part of broader strategies to lower the prevalence of NCDs [3, 4]. Elevated salt intake is directly linked to higher blood pressure, and in many countries, including Vietnam, average salt consumption far exceeds the recommended limits set by the World Health Organization (WHO), which advocates for less than 5 g of salt per day [5]. Numerous studies have demonstrated that reducing salt intake is a highly cost-effective, and in some cases, cost-saving strategy for lowering blood pressure and reducing the risk of cardiovascular diseases (CVDs) [3, 6, 7]. As a result, salt reduction has been identified as a priority intervention for preventing NCDs [8].

Vietnamese diets are often high in sodium due to salt, fish sauce, soy sauce, and other sodium-based condiments deeply rooted in cultural practices and traditional food preservation methods [9]. Recognizing the adverse health impacts of excessive salt consumption, Vietnam has implemented national policies to reduce sodium intake [10]. However, despite these efforts, there remains limited information on how salt consumption has changed over time among the general population, especially among individuals with NCDs such as hypertension, diabetes, and overweight. This lack of data is concerning, as understanding salt consumption trends in the general population and high-risk groups is crucial for monitoring the effectiveness of public health interventions designed to reduce sodium intake. With accurate and up-to-date information, assessing the success of these initiatives becomes easier. More importantly, excessive sodium intake can exacerbate conditions such as hypertension and insulin resistance, which are critical factors in the development and progression of diabetes [11, 12]. For individuals with diabetes, high salt intake is particularly problematic, as it can lead to elevated blood pressure, further complicating their health management [11]. Similarly, individuals with high body mass index (BMI) are often at increased risk for both hypertension and diabetes, creating a complex interplay between these conditions and dietary sodium intake [13]. By investigating salt consumption patterns in relation to these metabolic factors, targeted interventions can be developed to address the unique needs of these populations.

This study aims to fill the knowledge gap by examining changes in salt intake between 2015 and 2021, utilizing data from two national representative surveys conducted in Vietnam. The country has conducted three rounds of STEPwise approach to NCD risk factor surveillance (STEP surveys) in 2010, 2015, and 2021. These surveys offer nationally representative data on the Vietnamese population, making them efficient for investigating changes in many NCD risk behaviors over time. However, only the STEPs surveys from 2015 to 2021 included participants aged 18–69 and conducted spot urine tests to measure urinary sodium levels, a direct indicator of salt intake. This study used these two surveys to address three objectives: first, to assess changes in salt intake measuring by urinary sodium levels in the general population; second, to analyze this change among individuals with high-risk metabolic factors for NCDs, including hypertension, diabetes, and elevated body mass index (BMI); and finally assess the practices to reduce salt consumption among Vietnamese populations aged 18–69 in the year 2021. Understanding the salt consumption patterns of individuals with these risk factors is crucial for developing effective prevention and management strategies that can improve health outcomes.

## Methods

### Data Sources

This analysis used data from two STEP surveys in Vietnam. STEPs 2015, the final sample sizes for STEPs 1, 2, and 3 were 3,758 people aged 18–69 (with a 97.4% response rate), 3,036 people (78.7% response rate), and 2,816 people (73.0% response rate), respectively. In STEPs 2021, the sample sizes were 4,738 people aged 15 and over in STEP 1 (with a 94.76% response rate) and 3,712 people in STEPs 2 and 3 (with a 74.2% response rate). Information about these surveys was described elsewhere [14].

Both surveys employed a two-stage random systematic sampling approach, with Enumeration Areas as the primary sampling units. The sampling frame covered 15% of Vietnam’s population and included representation from all 63 provinces and cities. This analysis included only people aged 18–69 years old and with information on Urinary Sodium Levels from both STEPs (i.e., 2,893 people in the year 2015 and 3682 people in the year 2021).

### Measurement

STEPs were large-scale studies; therefore, spot urine collections were performed due to their simplicity, lower cost, and reduced participant burden [15]. When combined with validated estimation equations, spot urine provides reasonably accurate population-level sodium intake trends, making it suitable for public health surveillance [16]. It also avoids biases introduced by unrepresentative subsamples of 24-hour urine and ensures methodological consistency. While not ideal for individual assessments, spot urine effectively monitors dietary changes and informs policy interventions [15, 17].

In both 2015 and 2020, urine samples were collected following the same standardized protocol, in accordance with WHO guidelines, by Provincial Preventive Medicine Centers/Centers for Disease Control and Prevention (CDC) under the supervision of National and Regional Epidemiology Institutes/Pasteur Institutes. All samples were sent to the National Institute of Nutrition (NIN), where they were analyzed in the same laboratory using identical methods. Urinary sodium (Na) and potassium (K) concentrations were determined with Ion-Selective Electrodes (ISE) in an automated analyzer, following the same standard operating procedures (SOPs) in both years. The NIN laboratory adheres to strict quality assurance protocols and participates in external quality control programs to ensure measurement accuracy and reliability. By maintaining consistent methodologies, instrumentation, and quality control measures across both surveys, we minimized the risk of systematic measurement error and ensured that the observed decline in population sodium intake reflects a true trend rather than methodological differences.

### Study Outcomes

#### People’s 24-hour Salt Intake

We used the Intersalt Southern European equation [15] with the spot urine test to compute 24-hour sodium intake. This method has been proven to be suitable for estimating sodium intake at the population level [15–17], its balance between accuracy and convenience.

For males, the equation below

$$\left(20.861+0.45\times Naspot\left(\frac{mmol}{L}\right)\right)-3.09\times Crspot\left(\frac{mmol}{L}\right)+4.16\times BMI\left(\frac{kg}{m^{2}}\right)+0.22\times Age\left(year\right)$$

For females, the equation below

$$\left(21.98+0.33\times Naspot\left(\frac{mmol}{L}\right)\right)-2.44\times Crspot\left(\frac{mmol}{L}\right)+2.42\times BMI\left(\frac{kg}{m^{2}}\right)+2.34\times Age\left(year\right)-0.03\times {Age}^{2}\left(year\right)$$

Which Naspot (Sodium concentration in spot urine in mmol/L), Crspot (Creatinine concentration in spot urine in mmol/L), and BMI (Body Mass Index).

The total sodium intake was converted to salt intake (by multiplication of 2.54/1000*23) to estimate the 24-hour salt intake (in grams).

#### Highly High Daily Salt Intake

High salt intake was defined as 24-hour salt intake equal to or more than 5 g per day (using the WHO recommendation) [5]. However, as most of the Vietnamese population consumed more than 5 g per day and as the global mean salt intake of adults was 10.78 g/per day in the year 2019 [18], this study also created an indicator of highly high salt intake (i.e., defined as 24-h salt intake is greater than 10.78 g per day).

### Independent Variables

#### Demographic Characteristics

Age group was categorized as 15–24, 25–34, 35–44, 45–54, 55–64, and 65 and over; Geographic location was defined as urban or rural; and Marital status as single, married, and other. The wealth index was calculated using household data, which included ownership of items such as televisions, bicycles, and cars, as well as household characteristics like flooring type, source of drinking water, and sanitation facilities. This index was used as a proxy for wealth status, excluding asset-related factors. The wealth index score was divided into quartiles: the lowest (<25th percentile), second (25th–50th percentile), third (50th–75th percentile), and highest (>75th percentile). The index’s validity in assessing relative household wealth in Vietnam has been documented in prior studies [19].

#### NCD Metabolic Factors

Hypertension was defined as systolic blood pressure ≥140 mmHg and/or diastolic ≥90 mmHg or currently on medication for raised blood pressure. Diabetes was defined as capillary whole blood glucose ≥7.0 mmol/L or on medication for raised blood glucose. For BMI, two cut-off points were used: Overweight/obesity by Asian cut-off (BMI ≥ 23.0) [20] and Overweight/obesity (BMI ≥ 25).

#### Practice to Reduce Salt Intake and Receive Advice About Reducing Salt

In STEPs2021, respondents were interviewed directly about practices to reduce salt intake and whether they received information on low-salt eating. The questions had response options of “Yes,” “No,” and “No response.”

### Statistical Approach

The SVY procedure in STATA 18 was used to estimate the average 24-hour salt intake and the proportion of individuals consuming more than 10.78 g of salt per day, together with their 95% CI. All calculations accounted for survey weights. This study analyzed changes in salt consumption across subgroups based on demographic factors (age, gender, geographic location), socio-economic factors (education, occupation, wealth quartiles), and metabolic risk factors for non-communicable diseases (hypertension, diabetes, high BMI).

### Ethical Consideration

The paper used secondary data from the STEPS surveys in 2015 and 2021, with all identifying information removed. In each STEPS survey, participants gave informed consent before completing the questionnaires and tests.

## Results

### Characteristics of Study Populations

Table 1 presents the demographic and health characteristics of study samples with information about urinary sodium levels in the STEPs survey in 2015 and 2021 (i.e., participating in STEPs round 3). The sample grew from 2,893 in 2015 to 3,682 in 2021. The proportion of participants aged 55–65 increased while younger groups declined. In terms of people with metabolic risk factors for NCDs, the proportion of people with hypertension, diabetes, and obesity all increased over time.

**TABLE 1:** Characteristics of the study sample with data on urinary sodium level, STEPwise approach to non-communicable diseases risk factor surveillance, Vietnam, 2015 and 2021.

Factor	Year 2015	Year 2021
N	2,893	3682
Age group		
Less than 25	243 (8.4%)	189 (5.1%)
25–34	537 (18.6%)	519 (14.1%)
35–44	725 (25.1%)	845 (22.9%)
45–54	696 (24.1%)	897 (24.4%)
55–65	571 (19.7%)	1,014 (27.5%)
Over 65	121 (4.2%)	218 (5.9%)
Married		
Not married	294 (10.2%)	280 (7.6%)
Married	2,303 (79.6%)	2,963 (80.5%)
Divorced/Widower	296 (10.2%)	439 (11.9%)
Sex		
Men	1,256 (43.4%)	1815 (49.3%)
Women	1,637 (56.6%)	1867 (50.7%)
Urban		
Urban	1,288 (44.5%)	1747 (47.4%)
Rural	1,605 (55.5%)	1935 (52.6%)
Hypertension		
No	2,250 (77.8%)	2,382 (64.7%)
Yes	642 (22.2%)	1,300 (35.3%)
Diabetes		
No	2,717 (95.0%)	3296 (89.6%)
Yes	143 (5.0%)	381 (10.4%)
BMI ≥ 23		
No	2,397 (82.9%)	2,920 (79.3%)
Yes	496 (17.1%)	762 (20.7%)
BMI ≥ 25		
No	1,816 (62.8%)	2,162 (58.7%)
Yes	1,077 (37.2%)	1,520 (41.3%)

### Changes in the Average 24-hour Salt Intake Among the General Population Between the Years 2015 and 2021

Table 2 shows the average 24-hour salt intake changes between 2015 and 2021 for the general population, stratified by several demographic factors such as urban/rural residence, age group, gender, and wealth index. In 2015, the average salt intake was 9.42 g/day, which reduced significantly to 8.07 g/day by 2021 (t-test, p < 0.01). Salt intake decreased across all age groups between 2015 and 2021, with the younger age group (<25 years) presenting a more significant reduction (from 9.28 g/day in 2015 to 7.77 g/day in 2021, p < 0.01). Men had consistently higher salt intake than women in both years, but both men’s and women’s salt intake reduced significantly over time.

**TABLE 2:** Changes in the average 24-hour salt intake among the general population, STEPwise approach to non-communicable diseases risk factor surveillance, Vietnam, 2015 and 2021.

	Year 2015		Year 2021	
	Mean	95% CI	Mean	95% CI
All population	9.42	9.27–9.56	8.07	7.97–8.16
Urban	9.43	9.21–9.65	8	7.85–8.16
Rural	9.41	9.22–9.6	8.11	7.99–8.23
Age-group				
Less than 25	9.28	8.91–9.65	7.77	7.36–8.18
25–34	9.54	9.31–9.77	8.05	7.86–8.24
35–44	9.51	9.32–9.69	8.31	8.16–8.46
45–54	9.58	9.36–9.8	8.3	8.14–8.46
55–65	9.13	8.87–9.39	7.86	7.74–7.99
Over 65	8.82	8.33–9.3	7.15	6.84–7.46
Gender				
Men	10.53	10.34–10.71	9.04	8.92–9.16
Women	8.3	8.16–8.43	7.09	6.99–7.19
Wealth index				
Q1	9.43	9.17–9.7	7.91	7.73–8.09
Q2	9.29	9.08–9.5	8.08	7.88–8.27
Q3	9.33	9.08–9.59	8.15	7.86–8.45
Q4	9.48	9.23–9.74	8.09	7.9–8.28
Q5	9.56	9.29–9.83	8.1	7.91–8.29

### Changes in the Average 24-hour Salt Intake Among People With Hypertension, Diabetes, and Overweight/Obesity Between 2015 and 2021

Table 3 shows the average 24-hour salt intake changes between 2015 and 2021 among people with hypertension, diabetes, and overweight/obesity. Those with hypertension and overweight/obesity had a significantly higher intake in both years compared to those with normal blood pressure and normal BMI. For all groups, individuals with/without hypertension, individuals with/without diabetes, and individuals with/without overweight/obesity, salt intake decreased significantly between 2015 and 2021. Overall, salt intake decreased across all groups, with higher reductions seen among individuals with elevated BMI, hypertension, and diabetes.

**TABLE 3:** Changes in the average 24-hour salt intake among people with hypertension, diabetes, and overweight/obesity, STEPwise approach to non-communicable diseases risk factor surveillance, Vietnam, 2015 and 2021.

	Year 2015		Year 2021	
	Mean	95% CI	Mean	95% CI
Hypertension				
No	9.32	9.17–9.48	7.92	7.8–8.03
Yes	9.82	9.59–10.05	8.49	8.33–8.64
Diabetes				
No	9.42	9.27–9.57	8.06	7.96–8.16
Yes	9.24	8.88–9.61	8.11	7.82–8.4
BMI ≥ 23				
No	9.07	8.9–9.24	7.6	7.49–7.71
Yes	10.11	9.93–10.29	8.82	8.69–8.94
BMI ≥ 25				
No	9.25	9.09–9.4	7.8	7.7–7.91
Yes	10.35	10.12–10.59	9.16	8.95–9.37

### Prevalence of People With Hypertension, Diabetes, and Overweight/Obesity Consuming Extremely High Salt Intake Over Time

The proportion of the Vietnamese population aged 18–69 consumed more than the WHO-recommended daily limit of 5 g of salt was 99.89 (99.7; 100) in 2015 and 97.79 (97.09; 98.48) in 2021. Table 4 presents the % of individuals consuming more than the global means of daily salt intake (i.e., 10.78 g of salt per day in 2023). This figure significantly decreased from 24.88% (95% CI: 22.19; 27.57) in 2015 to 8.31% (95% CI: 6.99; 9.64) in 2021. People with hypertension in 2015 had a higher prevalence of excessive salt consumption (29.75%) compared to those without hypertension (23.74%). This difference narrowed in 2021, with hypertensive individuals still having higher consumption (12.14%) compared to non-hypertensive individuals (6.93%), but both groups experienced marked reductions. In 2015, people with diabetes (21.28%) had a lower prevalence of excessive salt consumption than non-diabetics (24.84%). The deduction in this figure between 2015 and 2021 was more significant for non-diabetics than people with diabetes. Whether using a cutoff point of BMI ≥23 or ≥25 to define overweight/obesity, individuals with higher BMI consistently show a greater prevalence of excessive salt consumption compared to those with normal BMI, even though there has been a significant decrease over time.

**TABLE 4:** Changes in the prevalence of people consuming more than the global mean of salt consumption (10.78 g per day), STEPwise approach to non-communicable diseases risk factor surveillance, Vietnam, 2015 and 2021.

	Year 2015		Year 2021	
	Proportion	95% CI	Proportion	95% CI
All population	24.88	22.19–27.57	8.31	6.99–9.64
Location				
Urban	26.25	22.05–30.45	8.77	6.79–10.75
Rural	24.15	20.7–27.61	8.05	6.3–9.8
Age-group				
Less than 25	25.23	18.11–32.34	10.45	4.35–16.55
25–34	28.59	23.45–33.73	6.12	3.83–8.42
35–44	24.71	20.63–28.78	8.84	6.4–11.28
45–54	22.56	18.32–26.79	10.3	7.64–12.97
55–65	21.76	17.52–25.99	7.29	5.46–9.11
Over 65	22.73	13.74–31.73	3.41	0.88–5.94
Gender				
Men	41.57	37.61–45.52	16.06	13.65–18.47
Women	7.96	5.97–9.95	0.48	0.03–0.93
Hypertension				
No	23.74	20.8–26.67	6.93	5.43–8.42
Yes	29.75	25.23–34.26	12.14	9.52–14.77
Diabetes				
No	24.84	22.02–27.66	8.2	6.78–9.62
Yes	21.28	13.16–29.4	9.64	5.88–13.41
BMI ≥ 23				
No	18.9	15.81–22.0	3.71	2.61–4.8
Yes	36.66	32.6–40.71	15.61	12.97–18.25
BMI ≥ 25				
No	21.79	18.89–24.69	5.34	4.17–6.51
Yes	41.76	36.24–47.28	20.52	15.89–25.16

### Practices to Reduce Salt Intake in 2021

The most common salt reduction behaviors included limiting processed food consumption (71.8%), avoiding eating out (68.5%), and restricting salt when cooking (60.5%). Additionally, using spices instead of salt (58.6%) and limiting salt at the table (57.0%) were also frequently adopted (see Table 5).

**TABLE 5:** Salt intake reduction practices among study sample, STEPwise approach to non-communicable diseases risk factor surveillance, Vietnam, 2021.

Practices (n = 3688)	Frequency	%
Limit consumption of processed foods		
Yes	2,648	71.8
No	1,032	28.0
Nonresponse	8	0.2
Look at the salt or sodium content on food labels		
Yes	1,257	34.1
No	2,423	65.7
Non response	8	0.2
Buy low salt/sodium alternatives		
Yes	1,669	45.3
No	2014	54.6
Non response	5	0.1
Use spices other than salt when cooking		
Yes	2,160	58.6
No	1,521	41.2
Non response	7	0.2
Avoid eating food prepared outside of a home		
Yes	2,526	68.5
No	1,155	31.3
Non response	7	0.2
Restrict adding salt when cooking		
Yes	2,230	60.5
No	1,451	39.3
Non response	7	0.2
Restrict adding salt on the table (dipping food to salt and/or adding salt to food)		
Yes	2,102	57.0
No	1,577	42.8
Non response	9	0.2
Restrict eating salty foods such as stew, fry		
Yes	1725	46.8
No	1952	52.9
Non response	11	0.3
Do other things specifically to control your salt intake		
Yes	328	8.9
No	3330	90.3
Non response	30	0.8

**FIGURE 1:**
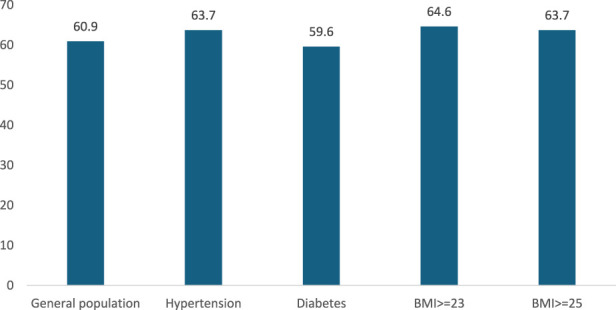
Percentage of people ever heard, seen or received advice about reducing salt, STEPwise approach to non-communicable diseases risk factor surveillance, Vietnam, 2021.

Less commonly practiced behaviors included looking at salt/sodium content on food labels (34.1%), buying low-sodium alternatives (45.3%), and restricting salty foods like stews or fries (46.8%) (Table 4). The proportion of people who ever heard, seen, or received advice about reducing salt among the general population was only 60.9%. This figure was not significantly higher among people with hypertension, diabetes, or overweight/obesity (Figure 1).

## Discussion

This study aimed to assess changes in salt intake among the Vietnamese population and to evaluate the effectiveness of salt reduction efforts. In 2021, the average 24-hour salt intake for Vietnamese individuals aged 18–69 was 8.07 g/day. While this is lower than the 2019 average for other Southeast Asian countries (9.8 g/day), it remains higher than other global regions such as the African Region or Eastern Mediterranean [18]. The findings also show a significant reduction in average salt intake in Vietnam, decreasing from 9.42 g/day in 2015 to 8.07 g/day in 2021. This suggests that public health efforts to reduce salt intake are making progress. The reduction in salt consumption was observed across different age groups, with the most notable decrease seen in individuals under 25 years old. These results are promising, given the well-established link between high salt intake and non-communicable diseases (NCDs), especially hypertension and cardiovascular diseases [1, 6, 11].

While salt consumption in Vietnam has decreased, further action is needed to meet health guidelines. In 2015, 99.89% of the population exceeded the WHO-recommended limit of 5 g of salt per day, dropping only slightly to 97.79% in 2021. Although the proportion consuming more than the global average of 10.78 g/day fell significantly from 24.88% to 8.31%, nearly the entire population still consumes excessive salt. The traditional Vietnamese diet includes salt as a staple, highlighting the need for stricter regulations on salt intake. While some countries have enforced mandatory limits on salt levels in food, Vietnam has predominantly relied on voluntary approaches [21]. However, previous studies suggest that voluntary programs are less effective in reducing salt consumption [4, 22]. Strengthening regulations could amplify the effectiveness of public health initiatives to reduce salt intake.

High-risk groups, such as individuals with hypertension, diabetes, and elevated BMI, consistently exhibit higher salt intake compared to others, even with noticeable reductions over time. In 2015, hypertensive individuals had a higher prevalence of excessive salt consumption (29.75%) compared to non-hypertensive individuals (23.74%), and while this gap narrowed by 2021, hypertensive individuals still consumed more salt (12.14% vs. 6.93%). Similarly, despite significant reductions, individuals with higher BMI (≥23 or ≥25) showed persistently higher salt intake than those with normal BMI. This trend emphasizes the vulnerability of overweight or obese populations to excessive salt intake, a key risk factor for cardiovascular diseases and other non-communicable diseases (NCDs). These findings highlight the need for more targeted interventions for high-risk groups, such as hypertensive and overweight individuals [6–8, 12]. Customized public health campaigns, stricter sodium content regulations in processed foods, and enhanced dietary education focused on these high-risk populations are crucial to further reducing salt intake and addressing the associated health risks [23].

The study also looked at salt reduction practices and found that the most common behaviors included limiting processed foods, avoiding eating out, and using less salt during cooking. These practices show a positive change in dietary habits among the population. However, less commonly adopted behaviors, such as checking food labels and buying low-sodium alternatives, indicate a need for more consumer awareness and knowledge about the sodium content in foods. Only 60.9% of the general population had heard, seen, or received advice about reducing salt intake. Surprisingly, this figure was not significantly higher among individuals with hypertension, diabetes, or overweight/obesity, who are at greater risk for complications related to high salt intake. This points to more targeted education and interventions for high-risk groups to encourage better adherence to salt reduction guidelines. Vietnam launched a National Plan for Dietary Salt Intake Reduction in 2018 and conducted a large-scale consumer awareness campaign. However, plans for sustaining and expanding these efforts remain uncertain due to limited resources [21]. Enhancing public knowledge through media and educational programs about the health risks associated with excessive salt intake and the benefits of adopting a low-salt diet may encourage more robust behavioral changes [24, 25].

This study benefits from a large, nationally representative sample (2,893 participants in 2015 and 3,682 in 2021), enhancing the generalizability of findings. The use of spot urine tests provides an objective and reliable measure of salt intake, minimizing biases associated with self-reported dietary data [15–17]. The repeated cross-sectional design (comparing 2015 and 2021) offers valuable insights into trends over time and the potential impact of public health efforts. Additionally, the study focuses on high-risk groups, including individuals with hypertension, diabetes, and overweight/obesity, adding depth to the analysis. The inclusion of salt reduction practices in 2021 further provides useful information on behavioral changes. The novelty of this study lies in its comprehensive analysis of salt intake trends within high-risk groups, providing more detailed insights into how these populations, who are more vulnerable to the health impacts of excessive salt, have responded to public health campaigns. While previous research has broadly examined national salt intake trends, this study stands out by specifically focusing on hypertensive, diabetic, and overweight/obese individuals, offering a deeper understanding of salt consumption patterns within these vulnerable populations. The study also employs the WHO-recommended STEPS framework [26], ensuring that the methodology aligns with international standards for measuring salt intake and enhancing the comparability of results. The inclusion of salt reduction practices further contributes to the novelty of this research, highlighting changes in behavioral practices and identifying gaps in consumer knowledge that could inform future public health strategies.

Despite these strengths, the study has several limitations. The cross-sectional nature of each survey round prevents tracking individual changes over time. Spot urine measurements, while practical and cost-effective, can be influenced by factors such as hydration status and physical activity, affecting individual accuracy [27]. The STEPS survey did not measure potassium alongside sodium, limiting the ability to assess the sodium-to-potassium balance, which is crucial for understanding cardiovascular health risks. A key limitation is the absence of 24-hour sodium excretion measurements for calibration in a representative subsample, which could lead to less precise sodium intake estimates, particularly among high-risk populations. This may impact the reliability of findings and hinder cross-country comparisons. Future studies should incorporate 24-hour urine collection in a representative subsample to improve the accuracy of sodium intake assessments. Although we did not have calibration, the STEPS survey is a standardized technical framework recommended by the World Health Organization (WHO) for monitoring salt intake over time [26]. This ensures methodological consistency and allows for meaningful comparisons within and across countries. Another limitation of this study is the absence of CVD outcome data in the STEPS survey, preventing a direct analysis of the relationship between high salt intake and CVD risks. However, we examined salt intake patterns among high-risk groups, including individuals with hypertension, diabetes, and overweight/obesity, to provide relevant insights. Finally, the cross-sectional design does not allow for causal inferences regarding the relationship between sodium reduction awareness, behavioral practices, and actual sodium intake. While a decline in population sodium intake was observed, it remains unclear whether this reduction resulted from individual efforts or broader policy initiatives. Future longitudinal or intervention studies are needed to better assess the effectiveness of specific sodium reduction strategies.

In conclusion, this study highlights a significant reduction in salt intake among the Vietnamese population from 2015 to 2021. However, the proportion of high-risk groups—those with hypertension, diabetes, and high BMI—who continue to consume excessive salt remains concerning. Ensuring these vulnerable groups are better informed and actively engaged in salt reduction efforts is crucial. Customized interventions focusing on education, community support, and policy reforms will be essential in reducing salt consumption and ultimately improving public health outcomes, especially for those at higher risk.
